# Identification of Lynch syndrome risk variants in the Romanian population

**DOI:** 10.1111/jcmm.13881

**Published:** 2018-10-16

**Authors:** Paul D. Iordache, Dana Mates, Bjarni Gunnarsson, Hannes P. Eggertsson, Patrick Sulem, Stefania Benonisdottir, Irma Eva Csiki, Stefan Rascu, Daniel Radavoi, Radu Ursu, Catalin Staicu, Violeta Calota, Angelica Voinoiu, Mariana Jinga, Gabriel Rosoga, Razvan Danau, Sorin Cristian Sima, Daniel Badescu, Nicoleta Suciu, Viorica Radoi, Ioan Nicolae Mates, Mihai Dobra, Camelia Nicolae, Sigrun Kristjansdottir, Jon G. Jonasson, Andrei Manolescu, Gudny Arnadottir, Brynjar Jensson, Aslaug Jonasdottir, Asgeir Sigurdsson, Louise le Roux, Hrefna Johannsdottir, Thorunn Rafnar, Bjarni V. Halldorsson, Viorel Jinga, Kari Stefansson

**Affiliations:** ^1^ deCODE genetics/AMGEN Reykjavik Iceland; ^2^ School of Science and Engineering Reykjavik University Reykjavik Iceland; ^3^ National Institute of Public Health Bucharest Romania; ^4^ Urology Department ‘Prof. Dr. Th. Burghele’ Clinical Hospital, University of Medicine and Pharmacy “Carol Davila” Bucharest Romania; ^5^ Department of Medical Genetics, Faculty of Medicine “Carol Davila” University of Medicine and Pharmacy Bucharest Romania; ^6^ Carol Davila University of Medicine and Pharmacy, Dr. Carol Davila Central University Emergency Military Hospital Bucharest Romania; ^7^ St. Mary” General Surgery Clinic University of Medicine and Pharmacy Carol Davila Bucharest Romania; ^8^ Carol Davila University of Medicine and Pharmacy Bucharest Romania; ^9^ Department of Pathology Landspitali University Hospital Reykjavik Iceland; ^10^ Faculty of Medicine School of Health Sciences, University of Iceland Reykjavik Iceland

**Keywords:** Colorectal cancer, Lynch syndrome, *MLH1*,*MSH2*,*MSH6, MUTYH*,*PMS2*, Romania

## Abstract

Two familial forms of colorectal cancer (CRC), Lynch syndrome (LS) and familial adenomatous polyposis (FAP), are caused by rare mutations in DNA mismatch repair genes (*MLH1*,*MSH2*,*MSH6*,*PMS2*) and the genes *APC* and *MUTYH*, respectively. No information is available on the presence of high‐risk CRC mutations in the Romanian population. We performed whole‐genome sequencing of 61 Romanian CRC cases with a family history of cancer and/or early onset of disease, focusing the analysis on candidate variants in the LS and FAP genes. The frequencies of all candidate variants were assessed in a cohort of 688 CRC cases and 4567 controls. Immunohistochemical (IHC) staining for *MLH1*,*MSH2*,*MSH6*, and *PMS2* was performed on tumour tissue. We identified 11 candidate variants in 11 cases; six variants in *MLH1*, one in *MSH6*, one in *PMS2*, and three in *APC*. Combining information on the predicted impact of the variants on the proteins, IHC results and previous reports, we found three novel pathogenic variants (*MLH1*:p.Lys84ThrfsTer4, *MLH1*:p.Ala586CysfsTer7, *PMS2*:p.Arg211ThrfsTer38), and two novel variants that are unlikely to be pathogenic. Also, we confirmed three previously published pathogenic LS variants and suggest to reclassify a previously reported variant of uncertain significance to pathogenic (*MLH1*:c.1559‐1G>C).

## 1 INTRODUCTION

Colorectal cancer (CRC) is the third most common cancer worldwide and the fourth most common cause of death from cancer,^1^ causing an estimated 8% of all cancer deaths. Lynch syndrome (LS) or hereditary nonpolyposis CRC is an autosomal dominant syndrome that accounts for about 1%‐3% of all CRC cases.^2^, ^3^ LS is the most common inherited cause of CRC and is caused by pathogenic germline mutations in one of four DNA mismatch repair (MMR) genes (*MLH1*,* MSH2*,* MSH6*, and *PMS2*).^4^ Carriers of LS mutations have an estimated 25%‐75% life‐time risk of CRC as well as increased risk of several other cancer types, including endometrial and ovarian cancer.^5^ Due to the low frequency of LS and heterogeneity in phenotypic expression, it has proven difficult to accurately establish population prevalence and to assess the penetrance of LS mutations.^6^

About 15% of CRC cases are somatically hypermutated as a consequence of MMR deficiency. Of these, 1%‐3% are due to LS while most of the remaining MMR‐deficient tumours have somatic inactivation of *MLH1* via hypermethylation of the gene promoter.^7^ In MMR‐deficient tumours, both copies of the same MMR gene have been inactivated, resulting in no production of the respective protein product. MMR‐deficient tumours exhibit several clinical characteristics that have implication for therapy, in particular with regard to the use of immune system modulators.^8^ Therefore, universal screening of CRC tumours, using microsatellite testing or immunohistochemistry (IHC) for the MMR proteins, has been recommended by several groups in Europe and the United States.^9^

To date, nothing is known about the prevalence of LS in the Romanian population and no mutations have been reported. The ROMCAN Project (“Genetic Epidemiology of Cancer in Romania”) started in 2012 with the major aim of characterizing genetic risk factors for CRC, breast cancer (BRC), prostate cancer (PrCa), and lung cancer (LuCa) in the Romanian population. The second goal of the ROMCAN Project is to define high‐risk groups for whom specific preventive measures can be implemented.

The objective of this study is to start assessing the impact of LS variants in CRC in Romania and provide a framework for screening in high‐risk families. Our present study was designed to identify high‐risk mutations in six CRC genes using whole‐genome sequencing (WGS). The frequencies of all candidate variants were then assessed in the entire ROMCAN cohort of 688 CRC cases and 4567 controls. In addition to the four MMR genes, we focused the analysis on mutations in two other CRC‐associated genes, *APC* and *MUTYH*.^10^

## 2 MATERIALS AND METHODS

### 2.1 Selection of CRC cases for WGS

We selected 61 CRC cases from the ROMCAN and ProMark projects sample collection, a hospital‐based sample set of 4567 cancer cases and controls recruited from five major hospitals in Bucharest between 2008 and 2017. The 61 CRC cases were selected using the following criteria: age at diagnosis lower than 40 years or family history of CRC, endometrial or gastrointestinal tumours. Two of the selected cases are of Roma origin. Both ProMark and ROMCAN are large‐scale genetic epidemiological studies investigating the profile of respectively PrCa and BRC, CRC, LuCa and PrCa in the Romanian population.

All subjects gave written informed consent prior to enrolment and accepted the use of personal and clinical data and biological samples for genetic research. The Bioethical Committee of the Medical School “Carol Davila” approved the study protocols. Trained interviewers performed face‐to‐face interviews, using standardized questionnaires, to collect personal data (ethnicity, marital status, education, height, and weight), lifestyle data (occupation, smoking history, coffee and tea consumption), and medical history (personal and familial). A description of relevant epidemiological and clinical information can be found in Table 1.

**Table 1:** Patient and tumour characteristics of the 61 CRC cases selected for whole‐genome sequencing

	N	%
Male	36	59.0
Female	25	41.0
TNM stage		
T0	3	4.9
Tis	3	4.9
T1	3	4.9
T2	3	4.9
T3	34	55.7
T4	4	6.6
T—NA	11	18.0
N0	26	42.6
N1	15	24.6
N2	9	14.8
NA—N	11	18.0
M0	45	73.8
M1	5	8.2
M—NA	11	18.0
SNOMED code		
M8480/3	2	3.3
M8140/3	59	96.7
ICD10 code		
C18.0	3	4.9
C18.2	5	8.2
C18.3	2	3.3
C18.4	5	8.2
C18.5	2	3.3
C18.6	6	9.8
C18.7	13	21.3
C18.8	1	1.6
C19.9	8	13.1
C20.9	16	26.2
Age at diagnosis		
30‐39 years	9	14.8
40‐49 years	16	26.2
50‐59 years	14	23.0
60‐69 years	15	24.6
70‐79 years	7	11.5

### 2.2 Whole‐genome sequencing and variant calling

DNA isolated from buccal samples from the 61 individuals was subjected to WGS to a targeted average depth of 30x. The samples were prepared following the TruSeq Nano sample preparation method and sequenced on Illumina HiSeq X machines. Sequencing reads were aligned to build 38 of the human reference sequence (GRCh38) using the Burrows‐Wheeler Aligner (BWA).^11^ Alignments were merged into a single BAM file and marked for duplicates using Picard.^12^ Only nonduplicate reads were used for the downstream analyses. Variants were called using version 3.8‐0 of the Genome Analysis Toolkit (GATK),^13^ using a multisample configuration.

### 2.3 Variant annotation and filtering

Variants were annotated using release 8.0 of the Variant Effect Predictor (VEP‐Ensembl).^14^ To filter out variants over a certain frequency threshold, we used a reference set of 38 000 Icelandic individuals whole‐genome sequenced at deCODE genetics, an extension of a previously described set of 15 220 WGS Icelanders.^15^ None of the variants described here had any carriers in the Icelandic dataset. Additional frequency filtering was performed using alleles from publicly available datasets of the Exome Aggregation Consortium.^16^

### 2.4 Genetic analysis

Only rare (below 1% allelic frequency) coding and splice region variants were considered, including variants with predicted high (stop, frameshift, and splice essential) and moderate (missense, in‐frame, and splice region) impact on protein function. We focused on single‐nucleotide polymorphisms and small indels (< 20 base pairs). We analysed pathogenic and expected pathogenic mutations in six genes, defined by the American College of Medical Genetics to be high‐risk genes in CRC: *MLH1*,* MSH2*,* MSH6*,* PMS2*,* APC*, and *MUTYH*.^17^

### 2.5 Frequency assessment in the Romanian population

All 11 coding variants found in the study were genotyped in the entire ROMCAN sample collection of 688 CRC cases, 254 BRC cases, 1457 PrCa cases, 1317 LuCa cases, and 1409 cancer‐free controls. The variants were genotyped using one of two assays: Centaurus^12^ or KASP.^13^ Sanger sequencing was used for one variant that failed in both assays. The primer sequences for the assays are listed in Table S1.

### 2.6 Immunohistochemistry

Paraffin blocks with tumour samples from all 11 carriers of variants in the LS genes were collected and sections from them stained for *MLH1*,* MSH6*,* PMS2* to assess if the protein was present. Immunohistochemistry was performed on 3 μm sections. Following deparaffinization in xylene, samples were rehydrated in ethanol and subjected to heat‐induced epitope retrieval (Tris/EDTA buffer, pH 9) in a 98.2°C water bath. Endogenous peroxidase activity was blocked with 3% hydrogen peroxide (DAKO). After incubation with the respective primary antibodies for 30 minutes at 20°C, EnVision FLEX Kit (DAKO ref:K8000) was used for detection.

Antibodies used were: Anti‐Human MutL Protein Homolog 1, Clone ES05, (DAKO ref:IR079), Anti‐Human Postmeiotic Segregation, Clone EP51 (DAKO ref:IR087, Anti‐Human MutS Protein Homolog 6, Clone EP49 (DAKO ref:IR086).^18^

## 3 RESULTS

Sequencing of the 61 CRC patients revealed 11 rare coding variants in CRC genes: six variants in *MLH1*, one variant in *MSH6*, one variant in *PMS2*, and three variants in *APC*. We examined literature and clinical trial submission data collected by ClinVar^19^ with respect to pathogenicity, six out of the 11 variants have been previously reported by ClinVar. All six previously reported variants have a frequency lower than 1% in the ExAC database.^16^

For each of the 11 variants, we assessed whether the variants had a high or moderate impact on protein function, based on the location of the mutation within its gene and its predicted molecular consequences (Table 2); frameshift, splice donor or acceptor and stop‐codon gain variants are predicted to have a high impact while in‐frame, missense and splice region variants are predicted to have a moderate impact. To assess the frequencies of the variants in the Romanian population, we genotyped all 11 variants in the ROMCAN cohort: 688 colorectal cases and 4567 cases with cancers other than CRC and controls (254 BRC cases, 1457 PrCa cases, 1317 LuCa cases, and 1409 cancer‐free controls) (Table 3). To test for loss of protein product, we stained MLH1, MSH6, and PMS2 in tumour tissue from paraffin blocks, collected from the carriers of the coding variants.

**Table 2:** Description of the 11 variants in CRC‐associated genes observed in the Romanian population

Nucleotide change	Ref	Alt	Exon	Position	Predicted protein effect	Consequence	ClinVar	InSIGHT	ACMG	Impact	Gene	IHC	APRP	Carriers in controls/carriers in CRC cases	Comment
NM_000179.2:c.3202C>T	C	T	5/10	chr2:47803449	NP_000170.1:p.Arg1068Ter	Stop‐gained	P	P	Tier II	High	MSH6	−	P	0/1	–
NM_000249.3:c.251_255delAACTG	AAACTG	A	3/19	chr3:37000997	NP_000240.1:p.Lys84ThrfsTer4	Frameshift	NA	NA	Tier II	High	MLH1	−	P	0/1	Novel
NM_000249.3:c.1148T>C	T	C	12/19	chr3:37025746	NP_000240.1:p.Met383Thr	Missense	LP	LP	Tier III	Moderate	MLH1	+	VUS	0/2	–
NM_000249.3:c.1559‐1G>C	G	C	intron	chr3:37040185	.	Splice acceptor	VUS	VUS	Tier II	High	MLH1	−	P	0/1	Suggest to classify as P
NM_000249.3:c.1755dupT	C	CT	16/19	chr3:37047540	NP_000240.1:p.Ala586CysfsTer7	Frameshift	NA	NA	Tier II	High	MLH1	−	P	0/1	Novel
NM_000249.3:c.2041G>A	G	A	18/19	chr3:37048955	NP_000240.1:p.Ala681Thr	Missense	P	P	Tier II	Moderate	MLH1	−	P	0/1	
NM_000249.3:c.2104‐6T>C	T	C	Intron	chr3:37050480	.	Splice region	NA	NA	Tier III	Moderate	MLH1	+	NP	2/1	Novel
NM_000038.5:c.2780C>G	C	G	16/16	chr5:112838374	NP_000029.2:p.Ala927Gly	Missense	VUS	NA	Tier III	Moderate	APC	NA	VUS	0/1	–
NM_000038.5:c.3682C>T	C	T	16/16	chr5:112839276	NP_000029.2:p.Gln1228Ter	Stop‐gained	P	NA	Tier II	High	APC	NA	VUS	0/1	–
NM_000038.5:c.5116T>A	T	A	16/16	chr5:112840710	NP_000029.2:p.Ser1706Thr	Missense	NA	NA	Tier IV	Moderate	APC	NA	VUS	0/1	Novel
NM_000535.5:c.630dupA	G	GT	6/15	chr7:5999182	NP_000526.1:p.Arg211ThrfsTer38	Frameshift	NA	NA	Tier II	High	PMS2	−	P	0/1	Novel

Ref, reference allele; Alt, alternative allele; exon, location of mutation/total number of exons; position, position of the variant in build38; ClinVar—ClinVar classification: P for pathogenic, VUS for variant of uncertain significance, LP for likely pathogenic, NP for not pathogenic and NA for not listed in ClinVar; InSIGHT—InSIGHT classification: P for pathogenic, VUS for variant of uncertain significance, LP for likely pathogenic, NP for not pathogenic and NA for not listed in InSIGHT; ACMG—classification based on the recommendations of the American College of Medical Genetics and Genomics, IHC—results of protein staining of the individuals tumour − means that the protein was not detected, + means that the protein was detected, APRP—Assessment of pathogenicity in the Romanian population represents our own assessment based on the ClinVar, InSIGHT, IHC results and predicted protein effect and is classified as follows: P for pathogenic, NP for not pathogenic, VUS for variant of uncertain significance; comment—this indicates the status of the variant compared to ClinVar and InSIGHT reports.

**Table 3:** Frequencies of the 11 variants in CRC‐associate genes observed in the Romanian population

Position in build 38	Predicted protein effect	Reference allele/alternative allele	N carriers in controls genotyped	N controls genotyped	N carriers in lung cancer cases genotyped	N lung cancer cases genotyped	N carriers in breast cancer cases genotyped	N breast cancer cases genotyped	N carriers in prostate cancer cases genotyped	N prostate cancer cases genotyped	N carriers in colorectal cancer cases genotyped	N colorectal cancer cases genotyped	N alleles in EXAC/total alleles in EXAC^a^
chr2:47803449	NP_000170.1:p.Arg1068Ter	C/T	0	1388	0	1148	0	248	0	1446	1	655	18/121286
chr3:37000997	NP_000240.1:p.Lys84ThrfsTer4	AAACTG/A	0	1392	0	1143	0	239	0	1436	1	634	NA
chr3:37025746	NP_000240.1:p.Met383Thr	T/C	0	1357	1	1151	0	242	0	1439	1	616	NA
chr3:37040185	.	G/C	0	1396	0	1151	0	246	0	1447	2	654	NA
chr3:37047540	NP_000240.1:p.Ala586CysfsTer7	C/CT	0	1395	0	1140	0	239	0	1440	1	645	NA
chr3:37048955	NP_000240.1:p.Ala681Thr	G/A	0	1396	0	1149	0	243	0	1445	1	658	NA
chr3:37050480	.	T/C	2	1385	0	1138	0	241	0	1434	1	634	NA
chr5:112838374	NP_000029.2:p.Ala927Gly	C/G	0	1437	0	1148	0	243	0	1422	1	654	4/121082
chr5:112839276	NP_000029.2:p.Gln1228Ter	C/T	0	1385	0	1147	0	243	0	1443	1	642	NA
chr5:112840710	NP_000029.2:p.Ser1706Thr	T/A	0	1231	1	1082	0	244	0	1269	1	608	NA
chr7:5999182	NP_000526.1:p.Arg211ThrfsTer38	G/GT	0	1373	0	1104	0	237	0	1434	1	640	1/121410

a To our knowledge, none of the individuals included in the EXAC cohorts are reported to be cancer patients.

We assessed the pathogenicity of these variants in the Romanian population based on IHC results, predicted protein effect and annotation in ClinVar and InSIGHT databases (Table 2, APRP, assessment of pathogenicity in the Romanian population).

We divide our results into two categories; novel variants and previously documented variants. We summarized all reports regarding the pathogenicity of the previously reported variants in Table S2, using the output from the ClinVar database. An overview of personal and familial history of cancer for the 11 carriers is listed in Table 4.

**Table 4:** Description of clinical information for the 11 CRC patients with mutations in CRC‐associated genes

Patient	Variant	Sex	Age at diagnostic	ICD‐10 code	SNOMED code	Cancer Grade	TNM—T	TNM—N	TNM—M	Relative 1	Age at diagnostic relative 1	Cancer in relative 1	Relative 2	Age at diagnostic relative 2	Cancer in relative 2
1	MSH6:p.Arg1068Ter	Male	44	C18.3	M8140/3	2	T3	N2	M1	Grandfather	54	CRC	Uncle	40	BrC
2	MLH1:p.Lys84ThrfsTer4	Female	45	C18.4	M8140/3	2	T3	N1	M0	Sister	42	CRC	–	–	–
3	MLH1:p.Met383Thr	Female	60	C18.7	M8140/3	2	T3	N1	M0	Father	59	BC	Brother	63	CRC
4	MLH1:c.1559‐1G>C	Female	41	C18.6	M8140/3	2	T3	N1	M0	Father	49	CRC	Brother	42	CRC
5	MLH1:c.1559‐1G>C	Male	43	C18.2	M8140/3	2	T3	N0	M0	Sister	NA	CRC	–	–	–
6	MLH1:p.Ala586CysfsTer7	Male	65	C18.6	M8480/3	2	T3	N1	M0	Brother	37	CRC	–	–	–
7	MLH1:p.Ala681Thr	Male	47	C18.6	M8140/3	2	T3	N0	M0	Father	65	CRC	–	–	–
8	MLH1:c.2104‐6T>C	Female	50	C18.7	M8140/3	2	T3	N0	M0	Brother	55	CRC	–	–	–
9	APC:p.Ser1706Thr	Female	66	C18.0	M8140/3	2	T3	N0	M0	Father	64	CRC	Mother	79	CRC
9	APC:p.Ala927Gly	Female	66	C18.0	M8140/3	2	T3	N0	M0	Father	64	CRC	Mother	79	CRC
10	APC:p.Gln1228Ter	Male	58	C18.8	M8140/3	1	Tis	N0	M0	Father	52	CRC	–	–	–
11	PMS2:p.Arg211ThrfsTer38	Male	44	C18.7	M8140/3	1	T4	N0	M0	Father	64	GC	Brother	36	GC

Patient—number used in the paper for this individual, relative 1—the first relative with a neoplasic pathology reported by the patient, relative 2—the second relative with a neoplasic pathology reported by the patient.

CRC, colorectal cancer; BC, bone cancer; GC, gastric cancer; BrC, breast cancer.

### 3.1 Novel variants

#### 3.1.1 *MLH1*

*MLH1*: c.251_255delAACTG is a frameshift variant with predicted amino acid change Lys84ThrfsTer4, and consequently assessed as a high impact variant. IHC staining of the tumour of the carrier of this mutation revealed a loss of *MLH1* protein, indicating pathogenicity. We classify this variant as a pathogenic for LS based on the impact of the frameshift mutation and IHC results, classifying this variant as a tier II, level D based on the recommendations of the American College of Medical Genetics and Genomics (ACMG).

*MLH1*:c.1755dupT is a frameshift variant with predicted amino acid change Ala586CysfsTer7, and consequently annotated as a high impact variant. IHC staining of the patient's tumour revealed a loss of *MLH1* protein. Based on the impact of the frameshift mutation and IHC result, we classify this variant as a pathogenic for LS, as a tier II, level D based on the recommendations of the ACMG.

*MLH1*:c.2104‐6T>C is a splice region variant, and consequently annotated as having moderate impact. IHC staining of the patient's tumour did not reveal a loss of *MLH1* protein expression. Our results do not support that his variant should be classified as pathogenic. We classify this variant as a tier III based on the recommendations of the ACMG.

#### 3.1.2 *PMS2*

The frameshift variant *PMS2*:c.630dupA results in the predicted protein change Arg211ThrfsTer38, and consequently annotated as having high impact. IHC staining showed a loss of *PMS2* protein expression in the carrier of this variant. The patient was diagnosed at age 44 and had extensive family history of gastrointestinal tract cancers. Based on the impact of the frameshift mutation, and IHC, we classify this variant as pathogenic for LS, we classify it as tier II, level D based on the recommendations of the ACMG.

#### 3.1.3 *APC*

The missense variant *APC*:c.5116T>A results in a protein change of Ser1706Thr, and is consequently annotated as having moderate impact. It has not been previously reported either by ClinVar or other studies. According the clinical significance guidelines for classification of sequence variants, we consider this variant to be likely benign, we classify it as tier IV, based on the recommendations of the ACMG. The carrier of this mutation also had a previously reported *APC* variant of uncertain significance (VUS), *APC*:c.2780C>G (Ala927Gly) described below.

### 3.2 Previously documented variants

#### 3.2.1 *MSH6*

*MSH6*:c.3202C>T (RS63749843), is a stop‐gained variant, assessed as a high impact variant with a protein change of Arg1068Ter. This variant was previously reported as pathogenic in ClinVar by 10 different submitters involved in clinical testing and research. Tumour sample from the carrier of this variant had loss of *MSH6* protein expression, further supporting its pathogenicity. We classify this variant as a tier II, level D based on the recommendations of the ACMG.

#### 3.2.2 *MLH1*

*MLH1*:c.1148T>C (RS141344760) is a missense variant, and consequently of moderate predicted impact, resulting in a protein change of Met383Thr. IHC staining of the carrier's tumour showed positive protein expression for *MLH1*. The variant was previously reported as being of uncertain significance in ClinVar by five different submitters from clinical testing and research and our results do not support pathogenicity. We classify this variant as a tier III based on the recommendations of the ACMG.

*MLH1*:c.1559‐1G>C is a splice acceptor variant, annotated as high impact. This was the only candidate mutation found in two CRC cases. The tumour samples from both carriers had lost MLH1 protein and both had documented family history or CRC. The variant has been reported previously by two different submitters as likely pathogenic for LS and our results indicate that the variant is pathogenic. We classify this variant as a tier II, level D based on the recommendations of the ACMG.

*MLH1*:c.2041G>A (rs63750217) is a missense with a predicted protein change of Ala681Thr, and consequently of moderate impact. The tumour of the carrier had loss of *MLH1* protein staining. This variant was previously reported as pathogenic in ClinVar by nine different submitters from clinical testing and research. In addition, OMIM has classified the variant as pathogenic for LS II. We classify this variant as a tier II, level D based on the recommendations of the ACMG.

#### 3.2.3 *APC*

*APC*:c.2780C>G (rs587781500) results in the amino acid change Ala927Gly and consequently of moderate impact. It was previously reported as a VUS in ClinVar by four different submitters from clinical testing. We classify this variant as tier III based on the recommendations of the ACMG. As mentioned above, the carrier also had another likely benign variant in *APC*,* APC*:c.5116T>A (Ser1706Thr).

*APC*:c.3682C>T is a stop‐gained variant resulting in the protein change Gln1228Ter, and consequently of high impact. It was reported in ClinVar by a single submitter as pathogenic for familial multiple polyposis syndrome and was found in a recent study investigating somatic *APC* mutations and loss of heterozygosity status for 630 patients with sporadic CRC.^14^ We classify this variant as a tier II, level D based on the recommendations of the ACMG.

## 4 DISCUSSIONS

This study is the first assessment of rare variants underlying LS in CRC patients in Romanians. We identify new variants specific to the Romanian population and show that some variants previously reported to be pathogenic in other populations also occur in Romania.

We identified three novel pathogenic variants, two novel variants that are unlikely to be pathogenic. Also, we confirmed three previously published pathogenic variants and suggest to reclassify a variant previously classified as VUS as pathogenic. Due to study limitations, we were not able to classify the three *APC* variants identified in the Romanian population. We note that out of the two rare missense variants in APC identified in the same individual, we classify one as a likely benign variant based on ACMG's guidelines for classification of sequence variants.^20^ The other variant, p.Ala927Gly, has been reported previously as a VUS, but we note that it is located within a critical domain, intolerant to mutations. Our present study is the first one, to our knowledge, to examine rare sequence variants associated with CRC in the Romanian population.

In total, we identified six pathogenic variants, one nonpathogenic variant and four variants of uncertain significance in the Romanian population. In order to determine the prevalence of these variants in Romania, we assessed the frequencies of the 11 variants in the full ROMCAN cohort. As described in Table 3, none of the mutations were found in more than 1 CRC patients except for *MLH1*:c.1559‐1G>C. Our results do not suggest any strong association between the 11 variants identified here and BRC, LuCa or PrCa.

Identification of LS variants in the Romanian population is important in order to reduce the incidence and mortality of this multicancer disorder. Our present study is the largest effort, to our knowledge, to examine the genetic profile of this pathology in Eastern Europe. Due to study limitations, we were not able to extrapolate any other clinical observations, and we emphasize the need for future follow‐up studies in the Romanian population. This study is the first step towards improving our understanding of the genetic particularities of this pathology in Romania and provides new insights for the scientific community studying the genetic epidemiology of LS.

## CONFLICT OF INTEREST

The authors from deCODE genetics are employees of deCODE genetics/AMGEN.
