# Supplementary material for: Identification of Lynch syndrome risk variants in the Romanian population
Source: J Cell Mol Med. 2018 Oct 16;22(12):6068–76. doi: 10.1111/jcmm.13881 (PMC6237568; doi:10.1111/jcmm.13881)
Supplement: Supplementary file 1 [file JCMM-22-6068-s001.docx]

**Supplementary Table 1**: The primer sequence for the sequencing assays

| Position | Variant | Direction | Primer |
| --- | --- | --- | --- |
| chr5:112839276 | NM_000038.5:c.3682C>T | Forward 1 | TCAAAGAGTTCATCTGGACAAAGC |
| chr5:112839276 | NM_000038.5:c.3682C>T | Reverse 1 | CCTTTTGAGGCTGACCACTTCT |
| chr5:112839276 | NM_000038.5:c.3682C>T | Forward 2 | GCCACAGATATTCCTTCATCACA |
| chr5:112839276 | NM_000038.5:c.3682C>T | Reverse 2 | ACTCTGTGCAGAACTTGGATGG |
| chr5:112838374 | NM_000038.5:c.2780C>G | Forward 1 | CAGCCATTCATACCTCTCAGGA |
| chr5:112838374 | NM_000038.5:c.2780C>G | Reverse 1 | ATTTGGCATAAGGCATAGAACA |
| chr5:112838374 | NM_000038.5:c.2780C>G | Forward 2 | CAGATTGCCAAAGTCATGGAAG |
| chr5:112838374 | NM_000038.5:c.2780C>G | Reverse 2 | CATGTCCTATTTGAATTTTCCGACT |
| chr3:37000997 | NM_000249.3:c.251_255delAACTG | Forward 1 | GGGAATTCAAAGAGATTTGGAAAA |
| chr3:37000997 | NM_000249.3:c.251_255delAACTG | Reverse 1 | TCACCTCGAAAGCCATAGGTAGA |
| chr3:37000997 | NM_000249.3:c.251_255delAACTG | Forward 2 | TCAAGAAAATGGGAATTCAAAGAG |
| chr3:37000997 | NM_000249.3:c.251_255delAACTG | Reverse 2 | GGCTAAATCCTCAAAGGACTGC |
| chr3:37040185 | NM_000249.3:c.1559-1G>C | Forward 1 | GCTCAATTCAGGCTTCTTTGCT |
| chr3:37040185 | NM_000249.3:c.1559-1G>C | Reverse 1 | ACGAAGGAGTGGTTATGCAACA |
| chr3:37040185 | NM_000249.3:c.1559-1G>C | Forward 2 | TGAAGTGGGGTTGGTAGGATTC |
| chr3:37040185 | NM_000249.3:c.1559-1G>C | Reverse 2 | GCTTGGTGGTGTTGAGAAGGTA |
| chr3:37047540 | NM_000249.3:c.1755dupT | Forward 1 | TTCTTGGGAATTCAGGCTTCAT |
| chr3:37047540 | NM_000249.3:c.1755dupT | Reverse 1 | CTTTGGGACCATCTTCCTCTGT |
| chr3:37047540 | NM_000249.3:c.1755dupT | Forward 2 | AATTCAGGCTTCATTTGGATGC |
| chr3:37047540 | NM_000249.3:c.1755dupT | Reverse 2 | CAAGTCCTTCTTTGGGACCATC |
| chr3:37025746 | NM_000249.3:c.1148T>C | Forward 1 | GGGGAGATGGTTAAATCCACAA |
| chr3:37025746 | NM_000249.3:c.1148T>C | Reverse 1 | GACTGGACAGGGGTTTGCTC |
| chr3:37025746 | NM_000249.3:c.1148T>C | Forward 2 | CAGACTTTGCTACCAGGACTTGC |
| chr3:37025746 | NM_000249.3:c.1148T>C | Reverse 2 | TGCAGAAATGCATCAAGCTTCT |
| chr3:37050480 | NM_000249.3:c.2104-6T>C | Forward 1 | ACCAAGTCTTTCCAGACCCAGT |
| chr3:37050480 | NM_000249.3:c.2104-6T>C | Reverse 1 | GTGTTCCACAGTCCACTTCCAG |
| chr3:37050480 | NM_000249.3:c.2104-6T>C | Forward 2 | CCAGGACACCAGTGTATGTTGG |
| chr3:37050480 | NM_000249.3:c.2104-6T>C | Reverse 2 | TTAGGAGGCAGAATGTGTGAGC |
| chr2:47803449 | NM_000179.2:c.3202C>T | Forward 1 | CGATGAAGCCTCACTTTTACCC |
| chr2:47803449 | NM_000179.2:c.3202C>T | Reverse 1 | GTAATGCAAGGATGGCGTGAT |
| chr2:47803449 | NM_000179.2:c.3202C>T | Forward 2 | CTGATAAAACCCCCAAACGATG |
| chr2:47803449 | NM_000179.2:c.3202C>T | Reverse 2 | GATGGCGTGATCCTTTAAGCTC |
| chr7:5999182 | NM_000535.5:c.630dupA | Forward 1 | GAAGTAACCGGCCATCACTACC |
| chr7:5999182 | NM_000535.5:c.630dupA | Reverse 1 | TGTATCATTTCAGCAGGCATCC |
| chr7:5999182 | NM_000535.5:c.630dupA | Forward 2 | CCCAAACACAGAGCCGATATTT |
| chr7:5999182 | NM_000535.5:c.630dupA | Reverse 2 | AGTATGCCAAAATGGTCCAGGT |
| chr5:112840710 | NM_000038.5:c.5116T>A | Forward 1 | CCATTCCTACAGAAGGCAGAAG |
| chr5:112840710 | NM_000038.5:c.5116T>A | Reverse 1 | GTGACTTTTCCCTTTGGGCATA |
| chr5:112840710 | NM_000038.5:c.5116T>A | Forward 2 | GATGAGGCTCAAGGAGGAAAAA |
| chr5:112840710 | NM_000038.5:c.5116T>A | Reverse 2 | TTTTTCACACGGAAAGGCTTGT |
| chr3:37048955 | NM_000249.3:c.2041G>A | Forward 1 | TTGAATTTCTTTGGACCAGGTG |
| chr3:37048955 | NM_000249.3:c.2041G>A | Reverse 1 | CAGTGTGCATCACCACTGTACC |
| chr3:37048955 | NM_000249.3:c.2041G>A | Forward 2 | GCCTCAGTAAAGAATGCGCTATG |
| chr3:37048955 | NM_000249.3:c.2041G>A | Reverse 2 | GAGATGGGCAAGTTTCATCTCC |
