# Supplementary material for: Identification of Lynch syndrome risk variants in the Romanian population
Source: J Cell Mol Med. 2018 Oct 16;22(12):6068–76. doi: 10.1111/jcmm.13881 (PMC6237568; doi:10.1111/jcmm.13881)
Supplement: Supplementary file 2 [file JCMM-22-6068-s002.docx]

**Supplementary Table 2**: ClinVar reports regarding the pathogenicity of the Romanian variants.

| Variant | Clinical significance (Last evaluated) | Review status  (Assertion method) | Collection method | Condition (Mode of inheritance) | Origin | Citations | Submitter - Study name | Submission accession |
| --- | --- | --- | --- | --- | --- | --- | --- | --- |
| MLH1:c.1148T>C | Uncertain significance | criteria provided, single submitter | clinical testing | not specified | germline | NA | GeneDx | SCV000149361.12 |
|  | (May 26, 2017) | GeneDx Variant Classification (06012015) |  |  |  |  |  |  |
|  | Uncertain significance | criteria provided, single submitter | clinical testing | Hereditary cancer-predisposing syndrome | germline | NA | Ambry Genetics | SCV000215984.2 |
|  | (Jul 6, 2015) | Ambry Autosomal Dominant and X-Linked criteria (10/2015) |  |  |  |  |  |  |
|  | Uncertain significance | criteria provided, single submitter | clinical testing | not specified | germline | NA | Laboratory for Molecular Medicine,Partners HealthCare Personalized Medicine | SCV000539643.1 |
|  | (Mar 31, 2016) | LMM Criteria |  |  |  |  |  |  |
|  | Uncertain significance | criteria provided, single submitter | clinical testing | Lynch syndrome | germline | NA | Invitae | SCV000543625.1 |
|  | (Nov 26, 2016) | Invitae Variant Classification Sherloc (09022015) |  |  |  |  |  |  |
|  | Uncertain significance | no assertion criteria provided | research | Ovarian cancer (Autosomal dominant inheritance) | germline | NA | CSER_CC_NCGL; University of Washington Medical Center - ESP 6500 variant annotation | SCV000190337.1 |
|  | (Jun 1, 2014) |  |  |  |  |  |  |  |
| MSH6:c.3202C>T | Pathogenic | reviewed by expert panel | research | Lynch syndrome | germline | Other citation | InSiGHT | SCV000108028.2 |
|  | (Sep 5, 2013) | Guidelines v1.9 |  |  |  |  |  |  |
|  | Pathogenic | criteria provided, single submitter | clinical testing | not provided | germline | NA | GeneDx | SCV000211314.11 |
|  | (Jun 29, 2017) | GeneDx Variant Classification (06012015) |  |  |  |  |  |  |
|  | Pathogenic | criteria provided, single submitter | clinical testing | Lynch syndrome I | germline | PubMed | Pathway Genomics | SCV000223782.1 |
|  | (Oct 30, 2014) | ACMG Guidelines, 2015 |  |  |  |  |  |  |
|  | Pathogenic | criteria provided, single submitter | clinical testing | Lynch syndrome | germline | NA | Invitae | SCV000253778.3 |
|  | (Jul 16, 2016) | Invitae Variant Classification Sherloc (09022015) |  |  |  |  |  |  |
|  | Pathogenic | criteria provided, single submitter | clinical testing | Hereditary cancer-predisposing syndrome | germline | NA | Vantari Genetics | SCV000267056.1 |
|  | (Oct 28, 2015) | ACMG Guidelines, 2015 |  |  |  |  |  |  |
|  | Pathogenic | criteria provided, single submitter | clinical testing | Hereditary cancer-predisposing syndrome | germline | NA | Color Genomics, Inc., | SCV000537682.1 |
|  | (Dec 2, 2015) | ACMG Guidelines, 2015 |  |  |  |  |  |  |
|  | Pathogenic | criteria provided, single submitter | clinical testing | Hereditary cancer-predisposing syndrome | germline | NA | Ambry Genetics | SCV000580090.1 |
|  | (Apr 12, 2017) | Ambry Autosomal Dominant and X-Linked criteria (3/2017) |  |  |  |  |  |  |
|  | Pathogenic | criteria provided, single submitter | clinical testing | Lynch syndrome | germline | PubMed | Department of Pathology and Laboratory Medicine,Sinai Health System - The Canadian Open Genetics Repository (COGR) | SCV000592625.1 |
|  | (Apr 13, 2015) | ACMG Guidelines, 2015 |  |  |  |  |  |  |
|  | Pathogenic | criteria provided, single submitter | clinical testing | not specified | germline | NA | ARUP Laboratories, Molecular Genetics and Genomics | SCV000604284.1 |
|  | (May 5, 2017) | ARUP Molecular Germline Variant Investigation Process |  |  |  |  |  |  |
|  | Pathogenic | no assertion criteria provided | research | not provided | unknown |  | Mayo Clinic Genetic Testing Laboratories,Mayo Clinic | SCV000257241.1 |
| MLH1:c.1559-1G>C | Likely pathogenic | reviewed by expert panel | research | Lynch syndrome | germline | Other citation | InSiGHT | SCV000106252.2 |
|  | (Sep 5, 2013) | Guidelines v1.9 |  |  |  |  |  |  |
|  | Likely pathogenic | criteria provided, single submitter | clinical testing | Hereditary nonpolyposis colon cancer | germline | NA | Invitae | SCV000625078.1 |
|  | (Feb 17, 2017) | Invitae Variant Classification Sherloc (09022015) |  |  |  |  |  |  |
| MLH1:c.2041G>A | Pathogenic | reviewed by expert panel | research | Lynch syndrome | germline | Other citation | InSiGHT | SCV000106491.2 |
|  | (Sep 5, 2013) | Guidelines v1.9 |  |  |  |  |  |  |
|  | Pathogenic | criteria provided, single submitter | clinical testing | Hereditary nonpolyposis colon cancer | germline | NA | Invitae | SCV000218745.5 |
|  | (Feb 28, 2017) | Invitae Variant Classification Sherloc (09022015) |  |  |  |  |  |  |
|  | Pathogenic | criteria provided, single submitter | clinical testing | Hereditary cancer-predisposing syndrome | germline | PubMed | Ambry Genetics | SCV000275301.3 |
|  | (Feb 2, 2017) | Ambry Autosomal Dominant and X-Linked criteria (3/2017) |  |  |  |  |  |  |
|  | Pathogenic | criteria provided, single submitter | clinical testing | Lynch syndrome II | unknown | PubMed | Counsyl | SCV000488784.1 |
|  | (Jun 16, 2016) | Counsyl Autosomal Dominant Disease Classification criteria (2015) |  |  |  |  |  |  |
|  | Likely pathogenic | criteria provided, single submitter | clinical testing | Lynch syndrome | germline | PubMed | Department of Pathology and Laboratory Medicine,Sinai Health System - The Canadian Open Genetics Repository (COGR) | SCV000592438.1 |
|  | (May 27, 2016) | ACMG Guidelines, 2015 |  |  |  |  |  |  |
|  | Pathogenic | criteria provided, single submitter | clinical testing | not provided | germline | NA | GeneDx | SCV000616784.1 |
|  | (Jun 12, 2017) | GeneDx Variant Classification (06012015) |  |  |  |  |  |  |
|  | Pathogenic | no assertion criteria provided | literature only | Lynch syndrome II | germline | PubMed | OMIM | SCV000038915.2 |
|  | (Jan 1, 2006) |  |  |  |  |  |  |  |
|  | Likely pathogenic | no assertion criteria provided | clinical testing | Lynch syndrome II | germline | NA | Bioscientia Institut fuer Medizinische Diagnostik GmbH,Sonic Healthcare | SCV000484931.1 |
|  | Uncertain significance | no assertion criteria provided | research | not specified | unknown |  | Mayo Clinic Genetic Testing Laboratories,Mayo Clinic | SCV000257082.1 |
| APC:c.2780C>G | Uncertain significance | criteria provided, single submitter | clinical testing | Hereditary cancer-predisposing syndrome | germline |  | Ambry Genetics | SCV000184243.4 |
|  | (Sep 18, 2017) | Ambry Autosomal Dominant and X-Linked criteria (3/2017) |  |  |  | NA |  |  |
|  | Uncertain significance | criteria provided, single submitter | clinical testing | Familial adenomatous polyposis 1 | germline | NA | Invitae | SCV000259601.4 |
|  | (May 15, 2017) | Invitae Variant Classification Sherloc (09022015) |  |  |  |  |  |  |
|  | Uncertain significance | criteria provided, single submitter | clinical testing | not specified | germline | NA | GeneDx | SCV000566009.3 |
|  | (Mar 19, 2015) | GeneDx Variant Classification (06012015) |  |  |  |  |  |  |
